# Supplementary material for: Multimorbidity, healthcare utilization and socioeconomic status: A register-based study in Denmark
Source: PLoS One. 2019 Aug 1;14(8):e0214183. doi: 10.1371/journal.pone.0214183 (PMC6675513; doi:10.1371/journal.pone.0214183)
Supplement: S1 Table — (DOCX) [file pone.0214183.s001.docx]

**Table**. Algorithms used to define the 16 conditions

| **No.** | **Condition** | **Defined as a physical or mental health condition in the study** | **ICD-10 from the Danish National Patient Register** | **Definition** |
| --- | --- | --- | --- | --- |
| 1 | Diabetes | Physical |  | All persons included in the Danish Diabetes Register where the inclusion date is before the population date (1/1 2012) [22] |
| 2 | Cancer | Physical | C00-C43 or C45-C97 | (DIAG)^1^ |
| 3 | Back pain | Physical | M40-54 | (DIAG)^1^ |
| 4 | Osteoarthritis | Physical | M15-M19 | (DIAG)^1^ |
| 5 | Osteoporosis | Physical | M80-M82and/or for persons aged 45 years and older contacts with the ICD-10 codes S22.0, S22.1, S32.0, S32.7, S32.8, S42.2, S42.3, S42.4, S42.7, S42.8, S42.9, S52.5, S52.6, S52.7, S52.8, S52.9, S62.0, S62.1, S72 | (DIAG)^1^ and/or (MEDICINE)^2^ all medicine prescriptions with either ATC: M05B, G03XC01, H05AA02, H05AA03 |
| 6 | Joint disease | Physical | M05, M06.0, M06.8, M07.0, M07.1, M07.3, M10.0, M10.9 | (DIAG)^1^ |
| 7 | Allergies | Physical | J30 except J30.0 | (DIAG)^1^ and/or (MEDICINE)^2^ all medicine prescriptions with either ATC: V01AA02, V01AA03; V01AA05, V01AA11; R01AC, R01AD, R06A, S01G, R01BA52. |
| 8 | Chronic obstructive pulmonary disease (COPD) | Physical | J40, J41, J42, J43, J44, J47, J96 | (DIAG)^1^ All patients of minimum 35 years of age at contact. And/or (MEDICINE1)^2^ all medicine prescriptions with either ATC: R03AC, R03AK, R03BA, R03BB, R03CC, R03DA, R03DC, V03AN01. And/or (SERVICE10)^3^ if patients have had a minimum of two lab services within the last 12 months. Lab services (80) 7113 (lung spirometer test), (80) 7121 (lung function test). MEDICINE and SERVICES are ignored if there are contacts with J45 or J46 diagnosis. |
| 9 | Dementia | Mental health | F00, G30, F01, F02.0, F03.9, G31.8B, G31.8E, G31.9, G31.0B | (DIAG)^1^ All patients of minimum 60 years of age at contact and/or (MEDICINE)^2^ all medicine with the prescriptions with the ATC N06D also for patients of minimum 60 years of age.  NB: Only one prescription (in contrast to two in the other algorithms) |
| 10 | Schizophrenia | Mental health | F20, F21, F22, F25, F28, F29, F31 | (DIAG)^1^ and/or (MEDICINE)^2^ all medicine prescriptions with either ATC: N05AX13, N05AX12, N05AH03, N05AX13, N05Ax08  NB: Only A-diagnosis not B-diagnosis |
| 11 | Long term use of antidepressants | Mental health |  | (MEDICINE) ^2^ At least three medicine prescriptions with the ATC N06A.  NB: At least three prescriptions (instead of two) on deferent dates within the latest five year with at least 730 days (2 years) between the first and the last one.  Patients with a schizophrenia diagnosis or with a dementia diagnosis are excluded from this algorithm. |
| 12 | Anxiety | Mental health | F40.1, F41.1 | (DIAG)^1^ and (MEDICINE) ^2^ At least three medicine prescriptions with the ATC N06A.  NB: At least three prescriptions (instead of two) on deferent dates within the latest five year with at least 730 days (2 years) between the first and the last one.  Patients with a depression diagnosis are excluded from this algorithm |
| 13 | High cholesterol | Physical | E78.0, E78.2, E78.4, E78.5 | (DIAG)^1^ and/or (MEDICINE)^2^ all medicine prescriptions with the ATC C10 |
| 14 | Hypertension | Physical | I10, I11, I12, I13, I15 | (DIAG)^1^ and (MEDICINE) ^2^ all medicine prescriptions with either ATC: C07B, C03A, C03B, C03E, C03X and/or (MEDICINE) ^2^ all medicine prescriptions with either ATC: C03C, C03D, C07A, C09 IF the person DOES NOT have hospital or outpatient contact with the ICD-10 codes I20.0, I21, I25.1, I50 and/or two medicine prescriptions with ATC: C08 IF the person DOES NOT have a hospital or outpatient contact with the ICD-10 codes I20-I25 |
| 15 | Stroke | Physical | G45, G46, I60, I61, I62, I63, I64, I65, I66, I67, I68, I69 | (DIAG)^1^ |
| 16 | Heart disease | Physical | I20, I21, I23, I24, I25, I50, I11, I13 | (DIAG)^1^ and/or (MEDICINE)^2^ all medicine prescriptions with either ATC: C01A, C01B, C01D, C01E |

^1^DIAG: All patients, any age unless specified otherwise, that have had a minimum of one hospital or outpatient contact, with one of the ICD-10 diagnosis codes of the condition within the last five years. Both ‘open’ (ongoing treatment) and ‘closed’ (finalized treatment) contacts, as well as primary (A), secondary (B) and additional (+) diagnosis are included.

^2^MEDICINE: All patients that have had a minimum of two medicine prescriptions as defined by the ATC codes and/or indication codes within a period of 24 months, at last one time during the specific period from the time of interest. MEDICINE1 indicates that medication criteria have been included from 1 year from the time of interest and MEDICINE2 for 2 years.

^3^SERVICE10: Patients that have had a minimum of one healthcare SERVICE during the last 10 years
