# Supplementary material for: Multimorbidity, healthcare utilization and socioeconomic status: A register-based study in Denmark
Source: PLoS One. 2019 Aug 1;14(8):e0214183. doi: 10.1371/journal.pone.0214183 (PMC6675513; doi:10.1371/journal.pone.0214183)
Supplement: S1 File — (DOCX) [file pone.0214183.s002.docx]

**Supporting Information**

**Acknowledgements**

We thank Jennifer Green for skillful editing.

**Ethics approval**

Approval to conduct the study was obtained from the Danish Data Protection Agency. No informed consent was required.

**Data sharing statement**

Due to restrictions related to Danish law and protecting patient privacy, the combined set of data as used in this study can only be made available through a trusted third party, Statistics Denmark. This state organisation holds the data used for this study. Danish scientific organisations can be authorized to work with data within Statistics Denmark and such organisations can provide access to individual scientists inside and outside of Denmark. Requests for data may be sent to Statistics Denmark: http://www.dst.dk/en/OmDS/organisation/TelefonbogOrg.aspx?kontor=13&amp;tlfbogsort=sektion. Additional requests can be sent to the authors with permission from Statistic Denmark, the Danish Health and medicines Authority, and the Research Centre for Prevention and health at the Capital Region of Copenhagen. Project number I-suite and ID number 03344 and BBH 2014-07.

**Funding**

This work was supported by grants for AF from the 50 million Crown Foundation from the Capital Region of Denmark.

The funders had no role in study design, data collection and analysis, decision to publish, or preparation of the manuscript.

**Competing Interests**

The authors have declared that no competing interests exist.
